# The Effects of Chromium Supplementation on Gene Expression of Insulin, Lipid, and Inflammatory Markers in Infertile Women With Polycystic Ovary Syndrome Candidate for *in vitro* Fertilization: A Randomized, Double-Blinded, Placebo-Controlled Trial

**DOI:** 10.3389/fendo.2018.00726

**Published:** 2018-11-28

**Authors:** Mehrnush Amiri Siavashani, Shahrzad Zadeh Modarres, Naghmeh Mirhosseini, Esmat Aghadavod, Saghar Salehpour, Zatollah Asemi

**Affiliations:** ^1^Preventative Gynecology Research Center, Shahid Beheshti University of Medical Sciences, Tehran, Iran; ^2^Laser Application in Medical Science Research Center, Shahid Beheshti University of Medical Sciences, Tehran, Iran; ^3^School of Public Health, University of Saskatchewan, Saskatoon, SK, Canada; ^4^Research Center for Biochemistry and Nutrition in Metabolic Diseases, Kashan University of Medical Sciences, Kashan, Iran

**Keywords:** chromium supplementation, gene expression, insulin metabolism, inflammatory markers, polycystic ovary syndrome

## Abstract

**Purpose:** This study was performed to determine the effects of chromium supplementation on the gene expression of insulin, lipid, and inflammatory markers in infertile women with polycystic ovary syndrome (PCOS) who were candidate for *in vitro* fertilization (IVF).

**Methods:** Forty women, aged 18–40 years, who had been selected for IVF were recruited in this randomized double-blinded, placebo-controlled trial. They (*n* = 20/group) were randomly assigned into intervention groups to take either 200 μg/day of chromium or placebo for 8 weeks. Inflammatory markers were measured at baseline and end of the trial. Genes related to insulin, lipid, and inflammation were expressed in peripheral blood mononuclear cells (PBMCs), using RT-PCR method.

**Results:** Chromium supplementation led to a significant reduction in serum high sensitivity C-reactive protein (hs-CRP) (−1.4 ± 1.5 vs. + 0.2 ± 2.2 mg/L, *p* = 0.01) compared with the placebo. RT-PCR findings indicated that chromium supplementation upregulated gene expression of peroxisome proliferator-activated receptor gamma (PPAR-γ) (*p* = 0.01), glucose transporter 1 (GLUT-1) (*p* = 0.001) and low-density lipoprotein receptor (LDLR) (*p* = 0.01), as well as downregulated gene expression of interleukin-1 (IL-1) (*p* = 0.004) in PBMCs of patients with PCOS compared with the placebo. Chromium supplementation had no significant effect on gene expression of IL-8, tumor necrosis factor alpha (TNF-α), transforming growth factor beta (TGF-β) and vascular endothelial growth factor (VEGF).

**Conclusion:** Overall, our findings demonstrated that infertile women with PCOS, who were candidate for IVF benefited from chromium supplementation for 8 weeks in terms of lowering hs-CRP and improving gene expression of PPAR-γ, GLUT-1, LDLR, and IL-1, though chromium had no effect on the gene expression of IL-8, TNF-α, TGF-β, and VEGF.

**Clinical Trial Registration Number:** http://www.irct.ir:IRCT20170513033941N32.

## Introduction

Polycystic ovary syndrome (PCOS), the major cause of anovulation and infertility among women of reproductive age, is the most common endocrine disorder affecting 5–10% of women worldwide (1). Accelerated lipolysis and subsequent dyslipidemia in women with PCOS is associated with metabolic abnormalities including obesity, insulin resistance and type 2 diabetes (T2DM) (2). The reflected changes in the follicular fluid (FF) of dominant follicle (3, 4) can directly affect the quality and metabolism of oocyte. Low-grade chronic inflammation is commonly blamed as an etiopathogenetic mechanism of infertility. Inflammatory cytokines are significantly higher among women with unexplained infertility (5). Pro-inflammatory factors have been shown to be significantly associated with higher failure rates of *in vitro* fertilization (IVF), especially in the implantation stage, among women with unexplained infertility (6). Studies have found elevated interferon gamma (IFN-γ) and interleukin-2 (IL-2) levels and decreased transforming growth factor beta (TGF-β) levels, an important endogenous anti-inflammatory mediator, (7) in the plasma of women with unexplained infertility compared with fertile controls during the implantation window (luteal phase) (8). Moreover, unexplained infertile patients have demonstrated elevated levels of serum IL-2, IL-4, IL-6, IL-21, tumor necrosis factor alpha (TNF-α), and IFN-γ, compared with fertile women (5).

The beneficial effects of chromium supplements have been already reported on metabolic abnormalities among women suffering from PCOS. In a meta-analysis conducted by Fazelian et al. (9), chromium supplementation decreased serum insulin and free testosterone and improved body weight in patients with PCOS. We have already published advantages of taking chromium supplements for 8 weeks on glycemic control, cardiometabolic risk parameters, and oxidative stress in infertile women with PCOS who were candidate for IVF (10). Further, chromium supplementation in diabetic rats showed anti-diabetic activities, which can be explained through chromium impact on the modulation of peroxisome proliferator-activated receptor gamma (PPAR-γ), insulin receptor substrate 1 (IRS-1) and nuclear factor κB (NF-κB) proteins (11). Similarly, Wang et al. (12) found that compared to obese control rats, those supplemented with chromium had significant improvement in their glucose disposal rates and an elevation in insulin-stimulated p-IRS-1 and phosphatidylinositol-3 kinase activity in skeletal muscles. In another animal experiment, chromium ingestion for 7 weeks, by diabetic rats, significantly decreased inflammatory markers including C-reactive protein (CRP), TNF-α and IL-6 (13).

Considering anti-diabetic and lipid-lowering effects of chromium (11, 14), we hypothesized that chromium supplementation might be beneficial in women with PCOS and candidate for IVF, who suffers from different metabolic abnormalities. According to our best knowledge, nothing has been published about the effects of chromium supplementation on gene expression of insulin, lipid, and inflammatory markers in infertile women with PCOS who were candidate for IVF. This trial was aimed to determine the effects of chromium supplementation on gene expression of insulin, lipid, and inflammatory markers in infertile women who suffered from PCOS and were candidate for IVF.

## Materials and methods

### Trial design and participants' characteristics

This randomized, double-blinded, placebo-controlled trial was registered by Iranian website for clinical trials registration (http://www.irct.ir:IRCT20170513033941N32). Forty infertile women with PCOS, aged 18–40 years, who were candidate for IVF without previous history of IVF, were included. This study was conducted in Taleghani Hospital, Tehran, Iran, between February and May 2018. Rotterdam criteria was used to confirm the diagnosis of PCOS (15). Participants with metabolic abnormalities including thyroid dysfunction, diabetes or impaired glucose tolerance were excluded from the study. This investigation was performed in accordance with the Declaration of Helsinki and Good Clinical Practice guidelines, and was approved by the research ethics committee of Shahid Beheshti University of Medical Sciences (SUMS), Tehran, Iran. Written informed consent was signed by all individuals.

### Intervention

Participants were randomly allocated into intervention groups to take either 200 μg/day chromium picolinate (Nature Made, California, USA) (*n* = 20) or placebo (Barij Essence, Kashan, Iran) (*n* = 20) for 8 weeks. Chromium supplements and placebos were matched in terms of shape, appearance, smell and packaging. Randomization was done using computer-generated random numbers. Randomization and allocation process were concealed from both the researchers and participants until the completion of final analyses. Another person, not involved in the trial and unaware of random sequences, assigned the subjects to the numbered bottles of tablets. Adherence to the supplements and placebos, throughout the trial, was monitored asking individuals to bring the medication containers back. To increase the compliance rate, all participants were receiving short daily reminder messages on their cell phones to take the supplements. Study participants completed dietary records at week 0, 4, and 8 of the intervention.

### Assessment of outcomes

Serum high sensitivity C-reactive protein (hs-CRP) levels were considered as the primary outcome and metabolic profiles were measured as the secondary outcomes. Fasting blood samples (15 mL) were collected at the beginning and end of the 8-week intervention at Taleghani Hospital (Tehran, Iran). Hs-CRP levels were measured using commercial ELISA kit (LDN, Nordhorn, Germany) with inter- and intra-assay coefficient variances (CVs) of lower than 7%. Plasma nitric oxide (NO) levels were measured using Griess method (16) with intra-assay CVs of < 5%.

### Isolation of lymphocyte cells

Blood samples were used to extract lymphocyte cells, using 50% percoll (Sigma-Aldrich, Dorset, UK). Cell count and viability test were conducted using trypan blue, RNA and, DNA extraction (17).

### RNA extraction and real-time PCR (RT-PCR)

RNA was extracted from blood samples using RNX-plus kit (Cinnacolon, Tehran, Iran). RNA suspension was frozen at −20°C until cDNA was derived. Following the extraction of total RNAs from each sample, RNA was quantified using UV spectrophotometer. Each sample OD 260/280 ratio of 1.7–2.1 represented no contamination with either protein or DNA (17). Using moloney murine leukemia virus reverse transcriptase (RT), isolated RNA was reverse transcribed to cDNA library. Gene expressions of PPAR-γ, glucose transporter 1 (GLUT-1), low-density lipoprotein receptor (LDLR), IL-1, IL-8, TNF-α, TGF-β, and vascular endothelial growth factor (VEGF) were conducted on mononuclear cells (PBMCs) of peripheral blood, using SYBR green detection and Amplicon Kit and applying quantitative RT-PCR and Light Cycler technology (Roche Diagnostics, Rotkreuz, Switzerland) (Table 1). Glyceraldehyde-3-phosphate dehydrogenase (GAPDH) primers were used as a housekeeping gene. Primers were designed using Primer Express Software (Applied Biosystems, Foster City, USA) and Beacon designer software (Takaposizt, Tehran, Iran). Relative transcription levels were calculated using Pffafi or 2^−ΔΔ*CT*^ methods.

**Table 1:** Specific primers used for real-time quantitative PCR.

**Gene**	**Primer**	**Product size (bp)**	**Annealing temperature (C)**
GAPDH	F: AAGCTCATTTCCTGGTATGACAACG	126	61.3
	R: TCTTCCTCTTGTGCTCTTGCTGG		
PPAR-γ	F: ATGACAGACCTCAGACAGATTG	210	54
	R: AATGTTGGCAGTGGCTCAG		
LDLR	F: ACTTACGGACAGACAGACAG	223	57
	R: GGCCACACATCCCATGATTC		
IL-1	F: GCTTCTCTCTGGTCCTTGG	174	56
	R: AGGGCAGGGTAGAGAAGAG		
IL-8	F: GCAGAGGGTTGTGGAGAAGT	150	56
	R: ACCCTACAACAGACCCACAC		
TNF-α	F: GTCAACCTCCTCTCTGCCAT	188	52
	R: CCAAAGTAGACCTGCCCAGA		
TGF-β	F: TTGAGACTTTTCCGTTGCCG	227	56
	R: CGAGGTCTGGGGAAAAGTCT		
VEGF	F: CTTCTGAGTTGCCCAGGAGA	216	54
	R: CTCACACACACACAACCAGG		

*GAPDH, glyceraldehyde-3-Phosphate dehydrogenase; IL-1, interleukin-1; IL-8, interleukin-8; LDLR, low-density lipoprotein receptor; PPAR-γ, peroxisome proliferator-activated receptor gamma; TNF-α, tumor necrosis factor alpha; TGF-β, transforming growth factor beta; VEGF, vascular endothelial growth factor*.

### Sample size and statistical methods

Sample size was calculated using the established formula for RCTs, with type one (α) and type two errors (β) were considered as 0.05 and 0.20 to provide the power of 80%. Based on a previous published study (18), a mean difference (d) of 1500.0 ng/mL and a standard deviation (SD) of 1496.1 ng/mL for hs-CRP were used to calculate sample size. Considering a probable dropout of 4 subjects in each intervention group, the final sample size was determined to be 20 (16 + 4) subjects per group.

The normality of data was assessed using Kolmogorov-Smirnov test. Analyses were performed using intention-to-treat (ITT) approach. Independent samples *t*-test was applied to determine the differences in anthropometric measures and gene expression of insulin, lipid and inflammation between intervention groups. The effects of chromium supplementation on inflammatory markers were assessed using repeated measures ANOVA test. Differences in proportions were evaluated by Fisher's exact test. The *P* < 0.05 were considered statistically significant. Statistical analyses were conducted using the Statistical Package for Social Science version 18 (SPSS Inc., Chicago, Illinois, USA).

## Results

**Figure 1:**
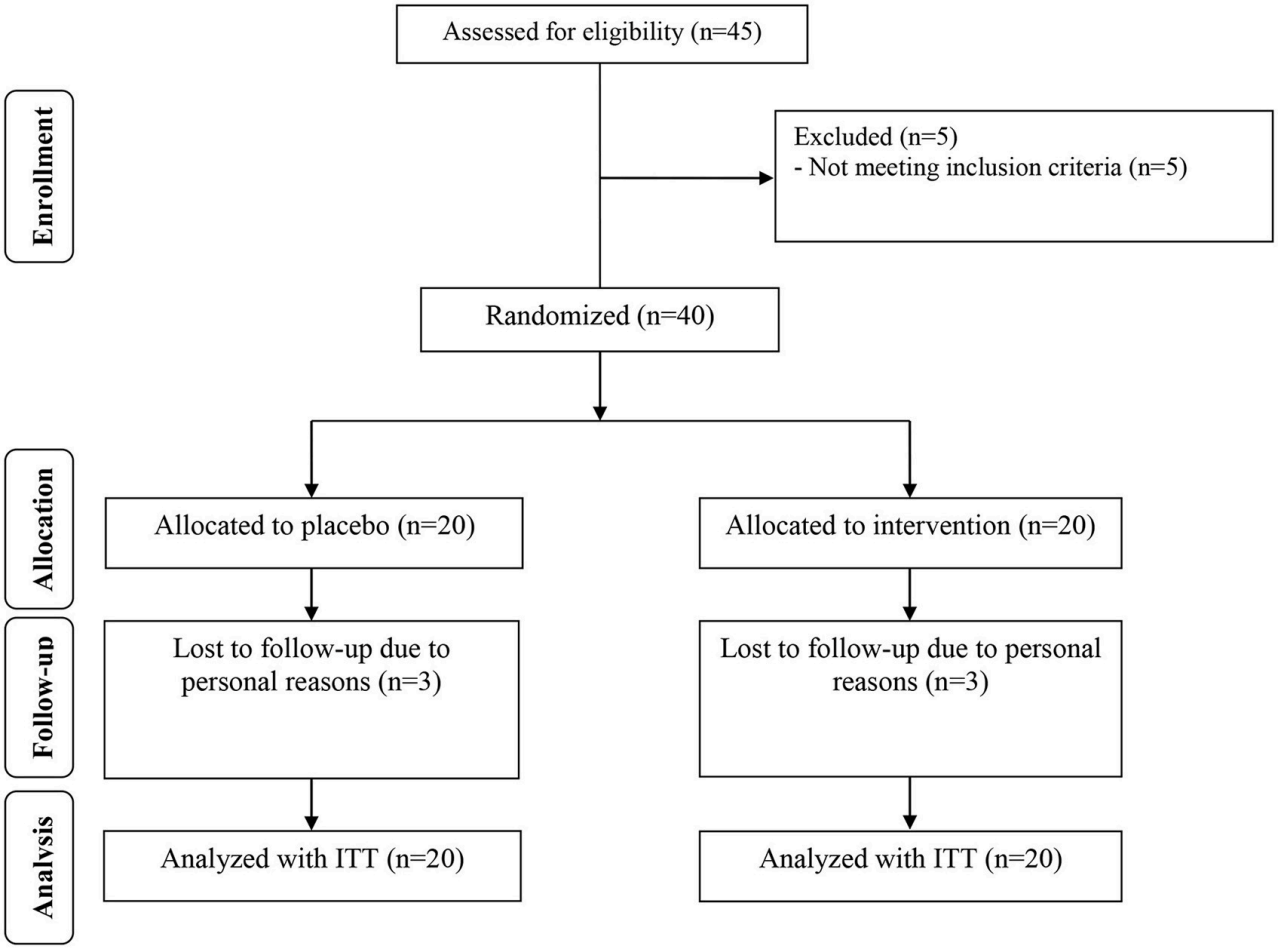
Summary of patient flow diagram.

Three dropouts were reported in each intervention group, due to personal reasons. So, overall 34 participants [infertile women with PCOS candidate for IVF receiving chromium (*n* = 17) and placebo (*n* = 17)] completed the study (Figure 1). Using ITT approach, all 40 participants (20 in each group) were included in the final data analysis. Compliance rate in this study was high, with more than 90% of tablets were consumed during the intervention in both groups. No side effects were reported following the consumption of chromium in women with PCOS in this study.

Anthropometric measures were not statistically different between intervention groups (Table 2). Moreover, following chromium supplementation, IVF outcomes including endometrial thickness, number of oocytes retrieved, number of fertilized oocytes, fertilization rate, pregnancy rate and number of embryo were not significantly different between supplemented and placebo groups.

**Table 2:** General characteristics of study participants.

	**Placebo group**	**Chromium group**	***P*^a^**
	**(*n* = 20)**	**(*n* = 20)**
Age (years)	33.8 ± 1.9	33.3 ± 2.7	0.55
Height (cm)	163.6 ± 1.9	164.2 ± 2.0	0.34
Weight at study baseline (kg)	72.2 ± 10.5	74.5 ± 7.0	0.41
Weight at end-of-trial (kg)	72.3 ± 10.4	74.5 ± 7.2	0.44
Weight change (kg)	0.1 ± 0.4	−0.02 ± 0.4	0.26
BMI at study baseline (kg/m^2^)	27.0 ± 3.9	27.7 ± 2.5	0.53
BMI at end-of-trial (kg/m^2^)	27.0 ± 3.9	27.6 ± 2.6	0.56
BMI change (kg/m^2^)	0.04 ± 0.1	−0.008 ± 0.1	0.25
**OUTCOMES OF IVF AT END-OF-TRIAL**			
Endometrial thickness (mm)	8.9 ± 1.2	9.4 ± 1.5	0.28
No. of oocytes retrieved (*n*)	16.5 ± 2.8	15.5 ± 1.9	0.20
No. of fertilized oocytes (*n*)	12.6 ± 2.5	13.6 ± 3.2	0.34
Fertilization rate, % (*n*)	58.8 (10/17)	64.7 (11/17)	0.72^†^
Pregnancy rate, % (*n*)	23.5 (4/17)	29.4 (5/17)	0.69^†^
No. of embryo (*n*)	9.8 ± 2.1	10.9 ± 2.4	0.15

*Data are means ± SDs*.

a *Obtained from independent t-test*.

† *Obtained from Fisher's exact test*.

*BMI, body mass index; IVF, in vitro fertilization*.

The average of macro- and micro-nutrient intakes was also not significantly different between two intervention groups throughout the treatment (Data not shown).

Chromium supplementation led to a significant reduction in serum hs-CRP (−1.4 ± 1.5 vs. + 0.2 ± 2.2 mg/L, *p* = 0.01), yet did not affect plasma NO levels (Table 3).

**Table 3:** Inflammatory markers at baseline and after the 8-week intervention in infertile polycystic ovary syndrome women candidate for *in vitro* fertilization that received either chromium supplements or placebo.

	**Placebo group (*****n*** = **20)**			**Chromium group (*****n*** = **20)**			***P*^a^**
	**Baseline**	**End-of-trial**	**Change**	**Baseline**	**End-of-trial**	**Change**
Hs-CRP (mg/L)	5.7 ± 1.8	5.9 ± 2.6	0.2 ± 2.2	4.8 ± 2.6	3.4 ± 1.7	−1.4 ± 1.5	0.01
NO (μmol/L)	41.8 ± 3.3	40.8 ± 4.2	−1.0 ± 3.5	44.8 ± 8.7	45.0 ± 9.7	0.2 ± 3.1	0.24

*Data are means ± SDs*.

a *Obtained from repeated measures ANOVA test*.

*Hs-CRP, high-sensitivity C-reactive protein; NO, nitric oxide*.

**Figure 2:**
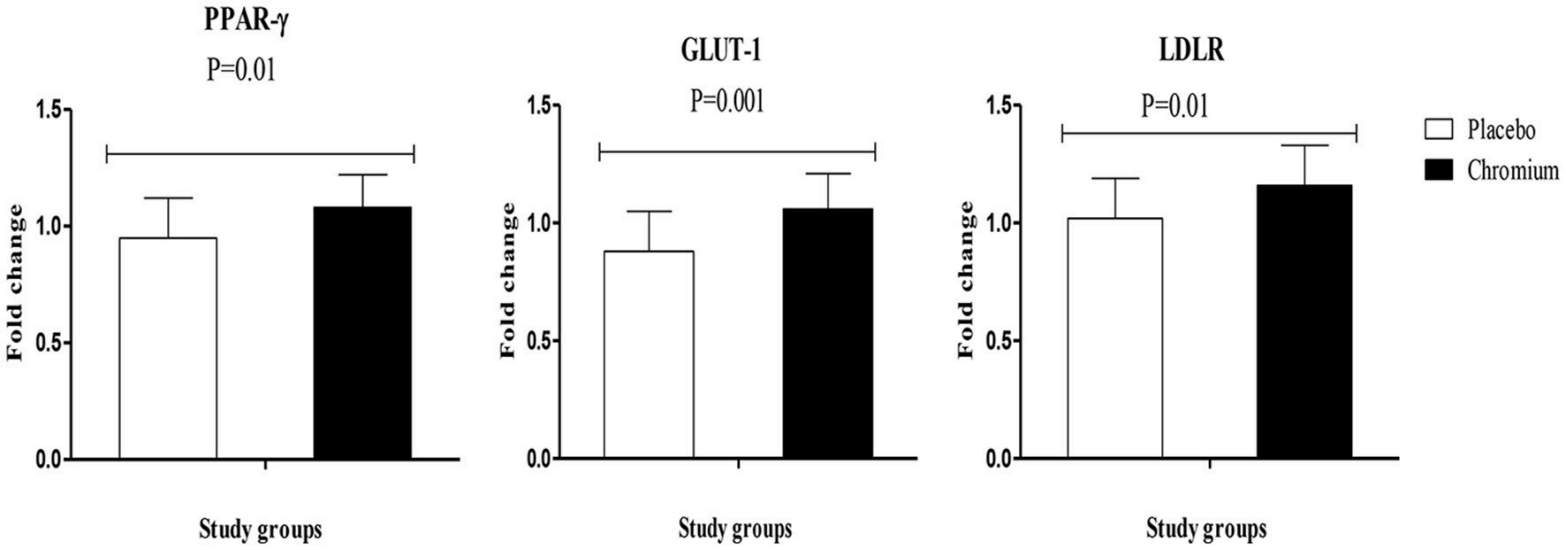
Fold change (means ± SDs) in gene expression levels of PPAR-γ, GLUT-1, and LDLR in women diagnosed with polycystic ovary syndrome who were candidate for *in vitro* fertilization, receiving chromium supplements and placebo. *P*-value was obtained from independent *t*-test. *N* = 20 in each group. IL-1, interleukin-1; IL-8, interleukin-8; LDLR, oxidized low-density lipoprotein receptor; PPAR-γ, peroxisome proliferator-activated receptor gamma; TNF-α, tumor necrosis factor alpha; TGF-β, transforming growth factor beta; VEGF, vascular endothelial growth factor.

RT-PCR findings indicated that chromium supplementation significantly upregulated the gene expression of PPAR-γ (*p* = 0.01), GLUT-1 (*p* = 0.001) and LDLR (*p* = 0.01) in PBMCs of patients with PCOS compared with the placebo (Figure 2).

**Figure 3:**
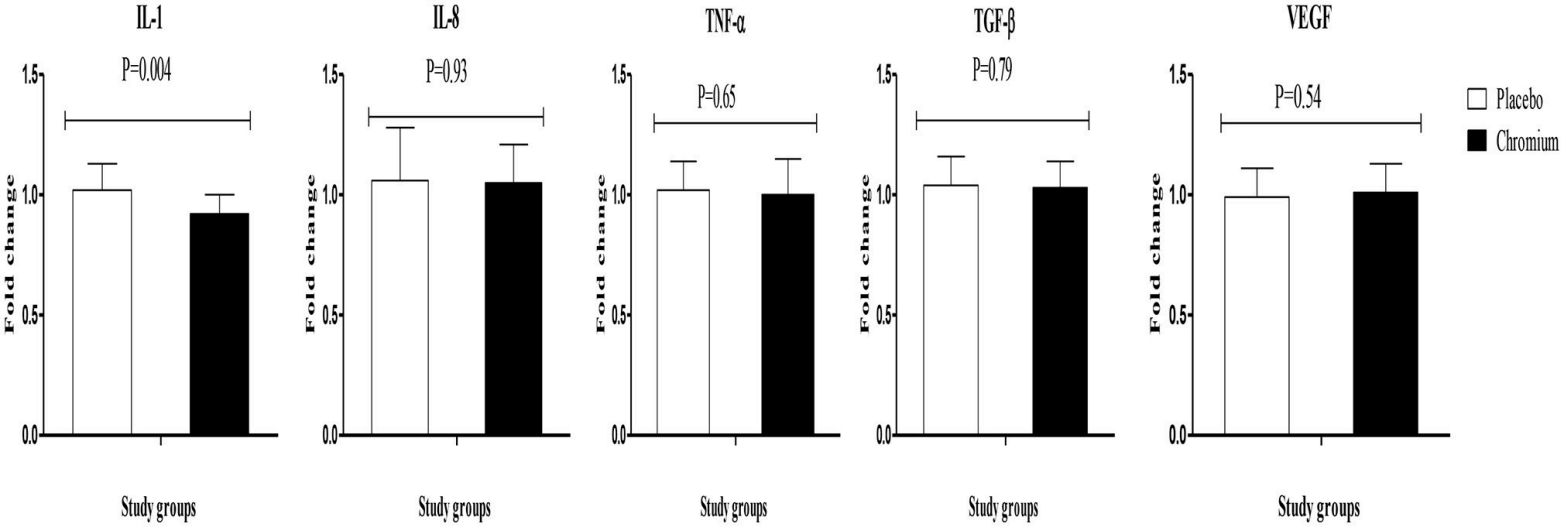
Change (means ± SDs) in gene expression levels of IL-1, IL-8, TNF-α, TGF-β, and VEGF in women diagnosed with polycystic ovary syndrome who were candidate for *in vitro* fertilization, receiving chromium supplements and placebo. *P-*value was obtained from independent *t*-test. *N* = 20 in each group. IL-1, interleukin-1; IL-8, interleukin-8; LDLR, oxidized low-density lipoprotein receptor; PPAR-γ, peroxisome proliferator-activated receptor gamma; TNF-α, tumor necrosis factor alpha; TGF-β, transforming growth factor beta; VEGF, vascular endothelial growth factor.

Chromium supplementation also downregulated the gene expression of IL-1 (*P* = 0.004) in PBMCs compared with the placebo (Figure 3). There was no significant effect of chromium supplementation on the gene expression of IL-8, TNF-α, TGF-β, and VEGF in PBMCs of patients with PCOS.

## Discussion

We found beneficial effects of chromium administration for 8 weeks in infertile women with PCOS who were candidate for IVF. Chromium supplementation lowered inflammation and improved gene expression of PPAR-γ, GLUT-1, LDLR, and IL-1, though it did not affect the gene expression of IL-8, TNF-α, TGF-β, and VEGF. To our best knowledge, this is the first trial reporting the effects of chromium supplementation on gene expression related to insulin, lipid, and inflammatory markers in infertile women diagnosed with PCOS and candidate for IVF.

### Effects of chromium on gene expression of insulin and lipid metabolism

Patients with PCOS are vulnerable to different metabolic abnormalities, such as increased insulin resistance, dyslipidemia and chronic inflammation (19, 20). The current study revealed that chromium supplementation for 8 weeks to infertile women with PCOS who were candidate for IVF significantly increased gene expression of PPAR-γ, GLUT-1, and LDLR. Consistent with our findings, chromium administration to diabetic rats showed anti-diabetic activities, probably related to the impact of chromium on the modulation of PPAR-γ and IRS-1 proteins (11). Wang et al. (12) also found that obese rats supplemented with chromium significantly increased their glucose disposal rates. Moreover, compared with obese controls, these rats showed an elevation in insulin-stimulated p-IRS-1 and phosphatidylinositol-3 kinase activity in skeletal muscles. In another study, chromium, biotin, or their combination significantly upregulated PPAR-γ expression in adipose tissue, which might have an effective insulin-sensitizing impact and reduce insulin resistance in a diabetic rat model (11). Chromium also can promote GLUT-4 trafficking and insulin-stimulated glucose transport (21). Likewise, rats with diabetic retinopathy who were supplemented with chromium for 12 weeks showed improvement in the gene expression of GLUT-1, GLUT-3, and insulin (22). PPAR-γ is the central regulator of insulin, glucose and lipid metabolism which is widely expressed in the adipose tissue. PPAR-γ might improve insulin sensitivity and lipid profiles in patients with type 2 diabetes as well as in diabetic rodent models (23). Current evidence suggests that PPAR-γ agonists may be beneficial in decreasing renal cortical lipids and proteinuria through a mechanism independent of their hypoglycemic impact (24, 25).

### Effects of chromium on gene expression of inflammatory markers

Our study supported that chromium administration for 8 weeks significantly decreased gene expression of IL-1 and serum hs-CRP levels in infertile women with PCOS who were candidate for IVF, though it did not affect gene expression of IL-8, TNF-α, TGF-β, and VEGF. Current evidence demonstrating the effects of chromium supplementation on the gene expression of inflammatory markers and their circulating levels are scarce and mostly limited to animal studies. Chromium supplementation for 8 weeks to Zucker diabetic fatty (ZDF) rats significantly decreased their serum CRP levels (26). In another study, chromium niacinate ingestion for 7 weeks reduced pro-inflammatory cytokines (TNF-α, IL-6, and CRP) levels, oxidative stress, and lipid concentrations in diabetic rats (13). Further, administration of a nutritional supplement containing chromium picolinate, phosphatidylserine, docosahexaenoic acid, and boron for 12 weeks significantly decreased CRP and TNF-α concentrations in rats fed high-fat diet (27). We found no significant effect of chromium supplementation on IL-8, TNF-α, TGF-β, and VEGF which might be explained through applying different study designs, type and dosages of chromium used, duration of intervention and participants' characteristics. Higher dosages of chromium may significantly improve the metabolic profiles including IL-8, TNF-α, TGF-β, and VEGF. The markers of inflammation and oxidative stress are elevated in many diabetic patients, which positively represents the vascular inflammation (28). Insulin resistance and subsequent vascular inflammation are the major risk factors for developing CVD (29). The suppressing effect of chromium on pro-inflammatory markers may be facilitated by inactivating oxidative stress pathways and reduction of Akt phosphorylation associated with the insulin resistance cascade in the liver (30). Current evidence has shown that increased inflammatory cytokines are involved in the outcomes of IVF. In a study conducted by Haimovici et al. (31), the women who gave live birth to their fetus had lower IL-6 levels. The inhibition of luteal cells producing progesterone in the presence of IL-1β may be one of the factors leading to the failure of the implantation (32). Increased follicular TNF-α and IL-6 might deteriorate the microenvironment in the follicle, thereby negatively affect the quality of oocyte and embryo (33). In another study conducted by Levin et al. (34), higher CRP levels, during IVF stimulation, were significantly associated with the failure of conception. According to the above-mentioned published evidence we assumed that the outcomes of IVF may be influenced by reduced inflammation. A recent study showed that using TNF-α inhibitor and intravenous immunoglobulin significantly improved the outcomes of IVF including; implantation, clinical pregnancy and live birth rates in young infertile women who had high levels of T helper 1/T helper 2 cytokines (35).

There were a few limitations in this study. Due to inadequate funding, we were not able to measure chromium levels and could not assess the effects of chromium on gene expression of oxidative stress.

Overall, our study suggests the advantages of chromium supplementation on lowering inflammatory markers through improving gene expression of PPAR-γ, GLUT-1, LDLR, and IL-1in infertile women with PCOS who were candidate for IVF.

## Author contributions

ZA helped in conception, design and statistical analysis of the manuscript. MA, SZ, NM, EA, and SS contributed in data collection and manuscript drafting. ZA supervised the study.

### Conflict of interest statement

The authors declare that the research was conducted in the absence of any commercial or financial relationships that could be construed as a potential conflict of interest.
